# Rare copy number variants in the genome of Chinese female children and adolescents with Turner syndrome

**DOI:** 10.1042/BSR20181305

**Published:** 2019-01-11

**Authors:** Li Li, Qingfeng Li, Qiong Wang, Li Liu, Ru Li, Huishu Liu, Yaojuan He, Gendie E. Lash

**Affiliations:** 1 Department of Obstetrics and Gynecology, Guangzhou Women and Children’s Medical Center, Guangzhou Medical University, 9 Jinsui Road, Guangzhou, Guangdong 510160, China; 2 Guangzhou Institute of Pediatrics, Guangzhou Women and Children’s Medical Center, Guangzhou Medical University, 9 Jinsui Road, Guangzhou, Guangdong 510160, China; 3 Department of Pediatrics Endocrinology, Guangzhou Women and Children’s Medical Center, Guangzhou Medical University, 9 Jinsui Road, Guangzhou, Guangdong 510160, China; 4 Prenatal Diagnostic Center, Guangzhou Women and Children’s Medical Center, Guangzhou Medical University, 9 Jinsui Road, Guangzhou, Guangdong 510160, China

**Keywords:** bone mineral status, copy number variation, CARD11 gene, Turner Syndrome

## Abstract

Turner syndrome (TS) is a congenital disease caused by complete or partial loss of one X chromosome. Low bone mineral status is a major phenotypic characteristic of TS that can not be fully explained by X chromosome loss, suggesting other autosomal-linked mutations may also exist. Therefore, the present study aimed to detect potential genetic mutations in TS through examination of copy number variation (CNV). Seventeen patients with TS and 15 healthy volunteer girls were recruited. Array-based comparative genomic hybridization (a-CGH) was performed on whole blood genomic DNA (gDMA) from the 17 TS patients and 15 healthy volunteer girls to identify potential CNVs. The abnormal CNV of one identified gene (*CARD11*) was verified by quantitative PCR. All cases diagnosed had TS based on genotype examination and physical characteristics, including short stature and premature ovarian failure. Three rare CNVs, located individually at 7p22.3, 7p22.2, and Xp22.33, where six genes (*TTYH3, AMZ1, GNA12, BC038729, CARD11*, and *SHOX* (stature homeobox)) are located, were found in TS patients. Quantitative PCR confirmed the CNV of CARD11 in the genome of TS patients. Our results indicate that *CARD11* gene is one of the mutated genes involved in TS disease. However, this CNV is rare and its contribution to TS phenotype requires further study.

## Introduction

Turner syndrome (TS) is a congenital disorder caused by partial or complete loss of one sex chromosome and occurs in approximately one in 3000–3500 female births [1,2]. The major phenotypic characteristics of TS include a reduction in growth velocity, short stature, primary ovarian insufficiency, low bone mineral status, and increased risk of fractures [3–5].

A single X chromosome (45,X0) is present in approximately half of all TS patients, while the others show mosaic patterns and structural derivatives including isochromosomes, rings, and deletions in sections of the genome [6–8]. It has been proposed that TS patients with a non-mosaic 45,X karyotype have a more severe phenotype of TS, compared with patients with 45,X,45,X/46,XX and 45,X/46,X,i(Xq) or 46,X,i(Xq) karyotypes [9]. However, the data correlating karyotype and phenotype is inconsistent with different studies reporting conflicting results [9–12].

Normally undetectable by standard karyotype tests, the examination of genomic copy number variations (CNVs) might be a more direct and objective method to specifically reveal structural variation, duplication or deletion of sections of the genome. Which section is repeated (and the number of repeats) or deleted, in the genome varies amongst individuals in the human population, and therefore may serve as a possible explanation for the variability in correlation between karyotype and phenotype. CNV examination can be performed through the analysis of array-based comparative genomic hybridization (a-CGH), which has recently emerged as an effective method to investigate autosome-linked loci that may play an important role in premature ovarian insufficiency (POI) [13].

We have recently demonstrated decreased bone mineral status in girls with TS [14]. The pathogenesis of this bone impairment in TS is unclear, and cannot be explained by X-linked chromosomal abnormalities alone. Therefore we sought to determine whether autosomal-linked CNVs were present in girls with TS.

## Materials and methods

### Ethics statement

The present study was approved by the Guangzhou Women and Children’s Medical Center Ethics Committee and conformed to the Declaration of Helsinki. Written informed consent was obtained from all patients included in the study (or their parents if the patient was younger than 18 years).

### Patients

Seventeen patients with TS and 15 healthy volunteer girls were recruited at the Departments of Pediatric Endocrinology and Gynecology Endocrinology at the Guangzhou Women and Children’s Medical Center, Guangzhou Medical University. None of the participants had been treated with growth hormone (GH). The girls (age >13 years) with primary amenorrhea had been treated by hormone replacement therapy (HRT). HRT treatment started from continuous low-dose conjugated estrogen therapy (0.5 mg daily for the first 6 months, continued with 1 mg daily for the following 6 months), and was then continued cyclically from the second year (conjugated estrogens 2 mg/day for 21 days, adding progesterone 10 mg/day for 10 days).

### Cytogenetic analysis

For each subject, cytogenetic analysis was performed on peripheral blood lymphocytes according to standard Giemsa stain G banding technology with 350–450 bands, more than 30 cells were karyotyped per patient [15].

### Anthropometry

In all subjects, standing height and weight were measured with a wall-mounted stadiometer and a mechanical balance. To ensure the most accurate results, height and weight measurements were repeated three times to obtain a mean result. Body mass index (BMI) was calculated as weight (kg) divided by height (m) squared.

### a-CGH analysis

All cases underwent a-CGH analysis. Genomic DNA (gDNA) was extracted from peripheral blood using Qiagen Mini Kits, according to the manufacturer’s protocol (Qiagen, MD, U.S.A.), then amplified and purified. Genome-wide high resolution CNV array CytoScan HD (Affymetrix, Beijing, China) was performed. Procedures for DNA digestion, ligation, PCR amplification, fragmentation, and labeling were performed according to the manufacturer’s protocols (Affymetrix).

The CytoScan® Array uses the Y-gender algorithm to determine a male/female call. The Y-gender algorithm uses a subset of Y probes which are then run through a Hidden Markov Model to determine copy number of 0 or 1, where 0 = female and 1 = male, if over 50 percent probes are female, then the software gives a female call to the sample, and *vice versa* for a male call. So in the condition of the X,0 patients, the sample is handled in the data analysis as female. In the case of mosaic XY/X,0 or Y chromosome partial deletion patients, the sample is handled as female or male based on the percentage of Y probes.

The sex chromosomes only share extensive homology with each other in the Pseudo Autosomal Regions (PARs). PAR1 is at the top of the p-arm and PAR2 at the bottom of the q-arm. Markers occurring in the PAR regions are mapped exclusively to the X chromosome. Therefore, in normal males the PAR regions of the X chromosome are expected to be CN = 2 (probes on the X and Y chromosomes both contribute to the signal), while the rest of the X chromosome is expected to be CN = 1 for normal males. As a result, we treat the two X PARs in males as independent units (CN = 2 expected) from the rest of the X chromosome (CN = 1 in males) when generating Copy Number Segments.

The reporting threshold of the sequence size of the CNVs was set at 100 kb with marker counting more than 50. Hybridization with the arrays was carried out with the Affymetrix cytogenetic 2.7 M arrays at 50°C for 16–18 h, using the Human Mapping Cytogenetic 2.7 M assay kit following the manufacturers’ standard protocol. The hapmap of 96 Asian individuals was used as a reference. Initial analysis and quality assessment of the array data were performed with a Genotyping Console (Affymetrix). The median absolute pairwise difference (MAPD) of each chip was used as a quality assessment of the array data. The median MAPD of this array was 0.26 and met the QC criteria of Affymetrix. To avoid missing CNV differences between patients and healthy volunteers, the results were analyzed by two software packages; Genotyping Console and Partek Genomics Suite, and the results merged. To minimize the potential false-positive rate from signal-to-noise ratio, only CNVs that involved at least 10 consecutive probe sets were considered, thus providing a median resolution of 7 kb. CNV classification was performed according to the Database of Genomic Variants (http://projects.tcag.ca/variation/): a CNV was defined as ‘rare’ or ‘common’ if it had never or previously reported in control subjects, respectively. The CNVs showing a significant difference between the genomes of patients and healthy volunteers were further confirmed by real-time PCR.

### Real-time quantitative PCR

To validate the two rare autosomal CNVs identified in the a-CGH analysis real-time quantitative PCR was performed. Fresh whole blood samples from patients 16 (analysis of TTYH3, AMZ1, and GNA12) and 17 (analysis of BC038729 and CARD11) as well as two age-matched healthy controls were taken and gDNA extracted using a blood genomic DNA extraction kit according to the manufacturer’s instructions (Generay Biotechnology, Shanghai, China). PCR was performed in triplicate in a 20-μl reaction volume containing 10 μl Go-Taq SYBR-Green PCR Master Mix (Applied Biosystems, Beijing, China), 10 µmol forward and reverse primers (Table 1; synthesized by Beijing Genomics Institute, Beijing, China), and 10 ng of gDNA. The reaction cycling conditions were 95°C for 2 min, followed by 40 cycles at 95°C for 15 s, and at 60°C for 30 s in an ABI StepOnePlus thermocycler (Applied Biosystems). After amplification, data were analyzed and exported by Sequence Detection Software (SDS) for the value of the threshold cycle (*C*_t_), differences in *C*_t_ values (Δ*C*_t_) between the test locus and the control locus (GAPDH), and the comparative *C*_t_ (ΔΔ*C*_t_) were used to calculate patient sample copy number value compared with the volunteer sample by relative quantitation (fold change).

**Table 1:** Primer sequences for PCR validation of array comparative genome hybridization identified CNVs

Gene	Forward	Reverse
*TTYH3*	CAGCCCCAGCAGTTTACAGA	CCCTCCACTGAGGTTTGGTC
*AMZ1*	ACGTACTGAACGCTTGCTGA	CAGCTACATTTGCAGGGGGA
*CNA12*	CCTTACAAGAGCCTGGTGGG	ATCCTCACCCTCTGAGGTCC
*BC038729*	GACCGAGATGATATGGCCCA	TGTCTGCAGTGTGGGATGAT
*CARD11*	GCAGAGCTGTTCTGGGATGT	GTGTTTCCCACTTCAACGCC
*GAPDH*	GAGTCAACGGATTTGGTCGT	GACAAGCTTCCCGTTCTCAG

### Statistical analysis

Fisher’s exact probability test was used to determine whether the rare autosomal CNVs identified in the current study were significantly different between TS patients and healthy volunteer girls, who all experienced normal spontaneous pubertal development.

## Results

### Genotype and phenotype of TS patients

All cases were diagnosed to be TS disease based on genotype examination and physical characteristics, including short stature and premature ovarian failure. The identified genotype and details of the heterosome abnormalities for each of the TS patients are listed in Table 2.

**Table 2:** Summary of genotype, body status, and bone mineral status of TS patients

Patient ID	Age (years)	Genotype	Del/Ins	Physical position	Chromosomal band	Size	Height (cm)	Weight (kg)	BMI (kg/m^2^)
P1	20	45X	Del	ChrX: 168546-155233731	Xp22.33-q28	155.07 Mb	135.7	30.8	16.8
P2	18	47,XY,+mar				155.07 Mb	139.2	41	21.2
P3	21	45,X[86]/46,X,i(X)(q10)[14]	Del	ChrX: 168546-155233731	Xp22.33-q28	155.07 Mb	138.4	33.1	17.6
P4	17	45X	Del	ChrX: 168546-155233731	Xp22.33-q28	155.07 Mb	148.6	56.5	32.3
P5	18	45X	Del	ChrX: 168546-155233731	Xp22.33-q28	2.50 Mb	141	38	18.5
P6	18	45X/46XX	Ins	ChrX: 201725-2703662	Xp22.33	65.05 Mb	148	40	18
			Del	ChrX: 90185870-155233731	Xq21.31-q28	155.07 Mb			
P7	12	45X	Del	ChrX: 168546-155233731	Xp22.33-q28	154.82 Mb	145.4	33.1	15.7
P8	11	45,X/46,XY	Del	ChrX: 168546-154985789	Xp22.33-q28	25.86 Mb	122.1	22.5	15.1
			Ins	ChrY:2915750-28775115	Yp11.31-q11.23	292 kb			
P9	5	45,X[30]/46,X,+mar[21]/46,XY[47]	Del	ChrX: 154941868-155233731	Xq28	118 kb	99.3	16	16.2
			Del	ChrY: 14432336-14549924	Yq11.21	5.15 Mb			
			Del	ChrY: 23653333-28799937	Yq11.223-q11.23	155.07 Mb			
P10	16	45,X[65]/47,XXX[35]	Del	ChrX: 168546-155233731	Xp22.33-q28	155.07 Mb	153.9	55.3	23.3
P11	20	45X	Del	ChrX: 168546-155233731	Xp22.33-q28	155.07 Mb	140.6	41	20.4
P12	16.7	45X	Del	ChrX: 168546-155233731	Xp22.33-q28	155.06 Mb	155.7	55.2	22.8
P13	13	45X	Del	ChrX: 168551-155233098	Xp22.33-q28	155.06 Mb	123.5	25.3	16.6
P14	13	45,X/46,XY	Del	ChrX: 168551-155233098	Xp22.33-q28	155.06 Mb	129.8	28.8	17.1
P15	10	45X	Del	ChrX: 168551-155233098	Xp22.33-q28	152.54 Mb	118.9	21.1	14.9
P16	4	45,X,SRY(+)	Del	ChrX: 26934660-155233098	Xp22.33-q28	11.22 Mb	97.1	15	15.9
			Del	ChrX: 2650424-13871751	Xp11.31-Xq11.21	155.06 Mb			
									
P17	6	45X	Del	ChrX: 168551-155233098	Xp22.33-q28	155.06 Mb	98.9	16	16.4
Total TS cohort (*n*=17)	14 ± 5.4						131.5 ± 18.9	33.5 ± 13.6	18.8 ± 4.3
Control cohort (*n*=15)	16 ± 3.9						160.0 ± 5.3	52.8 ± 5.0	16.0 ± 5.3

Abbreviations: Del, deletion; Ins, insertion.

### High-resolution a-CGH analysis detected rare CNV in X-linked and autosomal genes associated with TS

**Figure 1:**
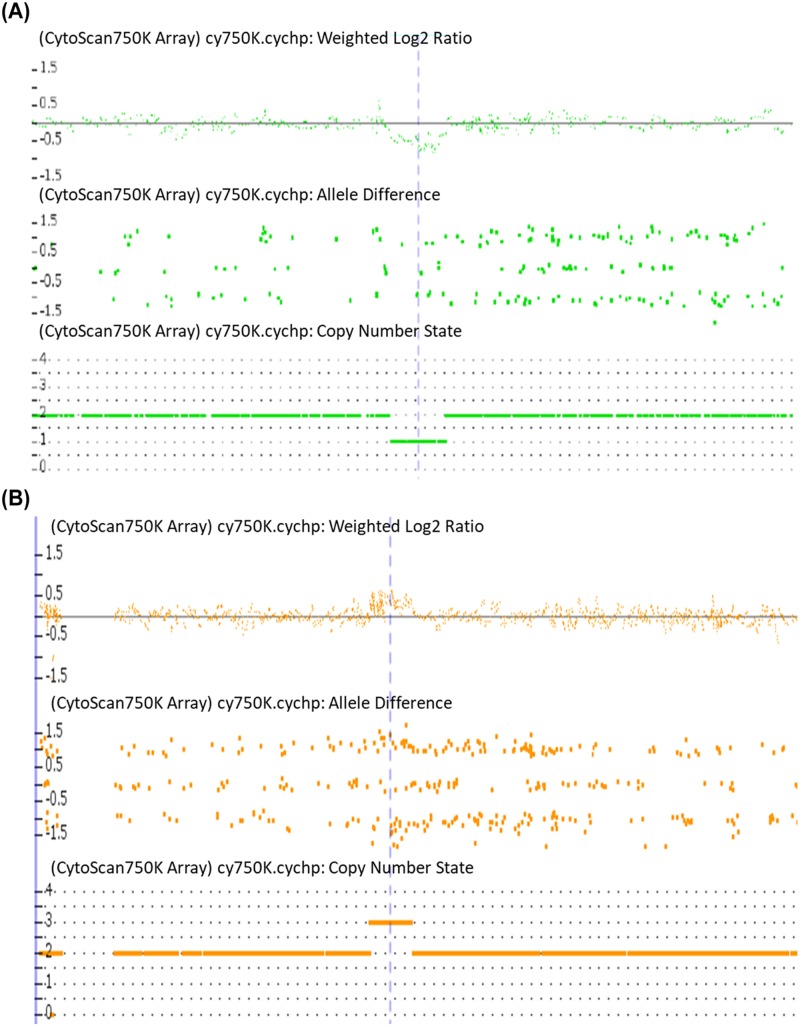

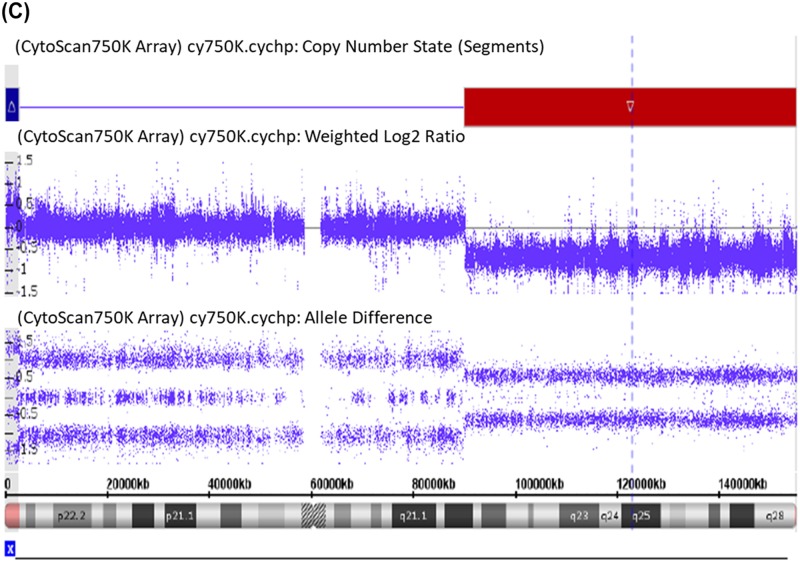
Rare CNVs identified by a-CGH in three Turners Syndrome patients (**A**) DNA copy number within 7p22.3 region offset down from normal baseline indicates sequence deletions detected by a-CGH in case 16. (**B**) DNA copy number within 7p22.2 region offset up from normal baseline indicates the sequence repeats detected by a-CGH in case 17. (**C**) DNA copy number within Xp22.33 region offset up from normal baseline indicates sequence repeats detected by a-CGH in case 6.

Through high-resolution a-CGH analysis on 17 TS patients, 18 CNVs were identified (Table 3). Two of them were rare and involved the genes *TTYH3, AMZ1*, and *GNA12* at 7p22.3, as well as the genes *BC038729* and *CARD11* at 7p22.2 (Figure 1A,B). In addition, in patient P6, a rare sequence repeat of at least 2.5 Mb including stature homeobox (*SHOX*) gene (ChrX:201725-2703662, hg19) at Xp22.33 was detected (Figure 1C); which revealed the presence of a structural abnormality of the second X chromosome that had not been identified previously by conventional cytogenetic analysis.

**Table 3:** Details of the identified CNVs in 17 TS cases

Patient ID	CNV^1^	Physical position^2^	Chromosomal band	Size	Genes included in the CNV^3^
Rare					
P16	Deletion	Chr7:2669219-2873356	7p22.3	204 kb	TTYH3, AMZ1, GNA12
P17	Insertion	Chr7:2966516-3334799	7p22.2	368 kb	BC038729, CARD11
P6	Insertion	ChrX:201725-2703662	Xp22.33	2.50 Mb	SHOX
Common					
P3	Insertion	Chr2:132058664-132269102	2q21.1	210 kb	WTH3DI, LOC389043, LOC401010
P4	Deletion	Chr6:66865606-66967579	6q12	102 kb	CRYBB2P1
P1	Deletion	Chr6:259519-381137	6p25.3	122 kb	DUSP22
P4	Deletion	Chr6:257339-381137	6p25.3	124 Kb	DUSP22
P12	Deletion	Chr6:254253-381137	6p25.3	127 Kb	DUSP22
P6	Insertion	Chr14:106206397-106709974	14q32.33	504 Kb	KIAA0125, ADAM6
P1	Insertion	Chr14:106253008-106761968	14q32.33	509 Kb	KIAA0125, ADAM6, LINC00226
P3	Insertion	Chr14:106251486-106750867	14q32.33	499 Kb	KIAA0125, ADAM6, LINC00226
P7	Insertion	Chr14:106227153-106717343	14q32.33	490 kb	KIAA0125, ADAM6
P10	Insertion	Chr14:106329183-106728149	14q32.33	399 kb	KIAA0125, ADAM6
P1	Deletion	Chr16:32564735-33814547	16p11.2	1.25 Mb	TP53TG3B, TP53TG3, TP53TG3C
P12	Deletion	Chr16:90050941-90155062	16q24.3	104 kb	PRDM7, FAM157C
P3	Insertion	Chr22:25656237-25922334	22q11.23-22q12.1	266 kb	IGLL3P, LRP5L, CRYBB2P1
P5	Insertion	Chr22:22929812-23258438	22q11.22	329 kb	POM121L1P, GGTLC2, MIR650
P7	Insertion	Chr22:23124497-23258438	22q11.22	134 kb	MIR650, IGLL5

1 CNVs classification has been performed according to the Database of Genomic Variants (http://projects.tcag.ca/variation/).

2 According to the genome assembly hg19 (UCSC Genome Browser, release February 2009, http://genome.cse.ucsc.edu, hg19).

3 Genes likely to be implicated in TS.

### Validation of the CNVs in patients 16 and 17

**Figure 2:**
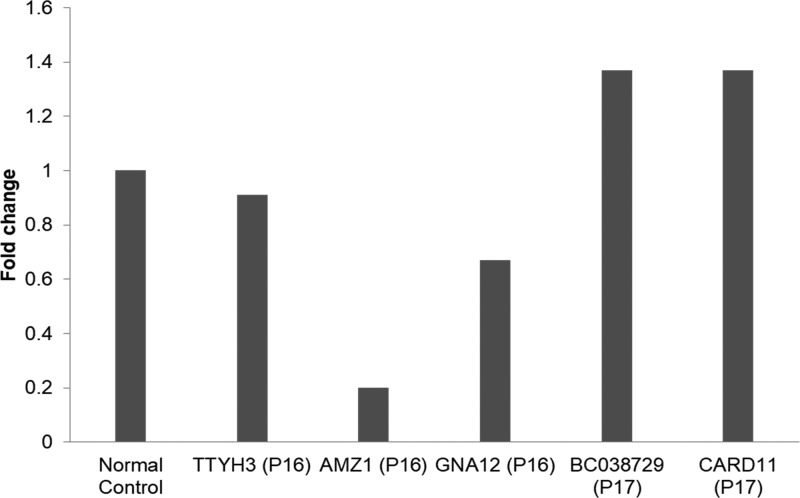
Fold change in levels of TTYH3, AMZ1, GNA12, BC038729, and CARD11 in the genome of P16 or P17 compared with an age-matched healthy control as determined by qPCR

The parents of P16 and P17 were examined by a-CGH and none had the same CNV as their daughters (data not shown), demonstrating that the CNVs observed in P16 and P17 were *de novo* mutations. For each of the genes in the validation study, two to three different pairs of primers were designed and tested, for four of the five genes there was good agreement across all pairs of primers, and therefore, for simplicity results from one pair are shown (Figure 2). These four genes were validated as having decreased (AMZ1 and GNA12) levels in P16 or increased (BC038729 and CARD11) levels in P17 compared with an age-matched healthy control. The fifth gene to be tested (*TTYH3*) was not validated to be decreased in P16 compared with an age matched healthy control.

## Discussion

In the present study, we detected three rare CNVs, involving six genes, in TS patients. The role of these genes in the pathogenesis of TS is currently unknown and warrants further research. The three rare CNVs detected covered a variety of genes including: TTYH3, AMZ1, GNA12, BC038729, CARD11, and SHOX.

TTYH3 belongs to the *tweety* family of genes encoding large-conductance chloride channels and are implicated in a wide array of cellular processes, such as cell division, cell adhesion, regulation of calcium activity, and tumorigenesis, particularly in neuronal cells throughout embryonic development [16]. GNA12 has been shown to be involved in human pancreatic adenocarcinoma cell migration, hepatocarcinoma progression, and oral squamous cell carcinoma invasion, and may play a role in the pathogenesis of pre-eclampsia [17–20]. *SHOX* gene plays roles associated with the development of physical stature. A deletion of SHOX is one of the known causes of Léri–Weill dyschondrosteosis and isolated short stature, while three copies of SHOX in cases with triple sex chromosome constitution are responsible for tall stature [21]. However, in our TS case of Patient P6 who has short stature (height: 148 cm), a rare sequence repeat of *SHOX* gene was found, which has not been reported previously. Based on current relevant literature, there is no reasonable explanation for why a perceived increased level of the *SHOX* gene would lead to short stature in this patient. Therefore, the role of the copy number variation of SHOX gene in the pathology of TS requires further study. In addition, to the best of our knowledge the functions of the AMZ1 and BC038729 genes have not yet been reported.

CARD11 is a member of the caspase recruitment domain family of proteins, which also shows homology with the membrane-associated guanylate kinase (MAGUK) family and can localize CARD–CARD association within the plasma membrane [22]. CARD11 was first identified by its ability to bind the NF-κB activator BCL10 and induce NF-κB activity [22]. CARD11 is a large multi-domain scaffold protein that transmits signals from both the T-cell receptor (TCR) and B-cell receptor to the IκB kinase (IKK) complex, which inducibly phosphorylates inhibitory I-κB proteins, leading to their ubiquitination and degradation and resulting in the stable nuclear translocation of NF-κB [23,24]. In a recent study, it was shown that RNF181 functions as an E3 ubiquitin ligase and inhibits antigen receptor signaling to NF-κB, downstream of CARD11, and can influence the signaling output of oncogenic CARD11 variants and the growth of CARD11-dependent human lymphoma cells [25]. A micro-duplication affecting only the CARD11 gene has previously been shown in a patient with immune deficiency [23,24,26–28]; in addition, it was also reported that decreased immunity may contribute to the progress of osteoporosis [29]. Therefore, we may speculate that increased CARD11 may contribute to diminished bone health in affected TS patients. Indeed, TS patients have abnormal immune alterations induced by epigenetic dysfunction, leading to the underlying immune deficiency of TS patient T cells during chronic otitis [30].

The genome-wide a-CGH approach with higher resolution, represents a valuable alternative clinical method for TS diagnosis. The a-CGH could indeed detect rare structural abnormalities of both the heterosome and the autosome in euploid cells, which were not identified by standard karyotyping, and therefore could precisely map the breakpoints, such as the repeat or the deletion of the sequence shown in the present study.

In conclusion, we demonstrate rare CNVs in three TS patients. One affected gene of interest is CARD11 which has been shown previously to be associated with phenotypes of immune deficiency [23], and that may also relate to diminished bone health. However, CNV of CARD11 has been identified only rarely. For example, a CARD11 mutation was observed in 5.5% of 173 mantle cell lymphoma (MCL) cases [31], its mutation copy was detected in 1 of 30 patients with premature ovarian failure (POF) [32], and none of the studies ever reported the association of CARD11 CNV with TS. Therefore, a much larger cohort study is required to determine the exact link between CARD11 CNV and TS pathogenesis. In addition, functional studies linking CARD11, immune deficiency, and bone mineral status are required.

## Abbreviations

a-CGH: Array-based comparative genomic hybridization
BCL10: B cell lymphoma 10
CN: Copy number
CNV: Copy number variation
*C*_t_: Tthreshold cycle
GAPDH: Glyceraldehyde 3-phosphate deydrogenase
gDNA: Genomic DNA
HRT: Hormone replacement therapy
IκB: Inhibitor of Nucelar factor kappa-light-chain-enhancer of activated B cells
MAPD: Median absolute pairwise difference
NF-κB: Nuclear factor kappa-light-chain-enhancer of activated B cells
PAR: Pseudo autosomal region
SHOX: Stature homeobox
TS: Turner syndrome
